# Responses of tree seedlings to understory filtering by the recalcitrant fern layer in a subtropical forest

**DOI:** 10.3389/fpls.2022.1033731

**Published:** 2022-11-24

**Authors:** Heming Liu, Mengfang Liang, Qingsong Yang, Jian Zhang, Guochun Shen, Zhenzhen Zhang, Xihua Wang

**Affiliations:** ^1^ Tiantong National Station for Forest Ecosystem Research, School of Ecological and Environmental Sciences, East China Normal University, Shanghai, China; ^2^ Department of ecology and climate, Shanxi Academy of Eco-Environmental Planning and Technology, Shanxi, China

**Keywords:** seedling establishment, ecological filter, biomass allocation, evergreen broad-leaved forest, *Diplopterygium glaucum*

## Abstract

The recalcitrant understory fern layer is an important ecological filter for seedling regeneration, yet how the fern layer influences seedling regeneration dynamics remains unclear. Here we transplanted 576 seedlings of four dominant tree species, *Castanopsis fargesii*, *Lithocarpus glaber*, *Schima superba* and *Hovenia acerba*, to the treatments of *Diplopterygium glaucum* retention and removal under an evergreen broad-leaved forest in eastern China. We monitored the survival, growth and biomass data of these seedlings for 28 months, and then used generalized linear mixed models to evaluate the treatment effects on seedling survival, growth, biomass and root-shoot ratio. Our results showed that fern retention significantly inhibited the seedling establishment of all four species. During the seedling development stage, the seedling relative growth rate of *L. glaber* decreased under fern retention, which was not the case for the other three species. Root-shoot ratio of *C. fargesii* and *L. glaber* increased significantly under fern retention. Our findings provide new evidence of the filtering effect of a recalcitrant fern understory. Notably, we observed that the response of tree seedlings to the recalcitrant fern understory was more sensitive in the establishment stage. Finally, our work highlights that the filtering effect of the recalcitrant fern understory changes depending on the regeneration stages, and that shade-tolerant species, *C. fargesii* and *L. glaber* were even more affected by fern disturbed habitats, suggesting that effective management should attempt to curb forest fern outbreaks, thus unblocking forest recruitment.

## Introduction

Tree seedling regeneration determines forest community dynamics and is a crucial component in forest restoration and management (Grubb, 1977; Royo and Carson, 2022), with symmetric and asymmetric interactions playing important ecological roles during seedling regeneration stages (Chesson, 2000; Comita et al., 2010; Johnson et al., 2012; Liu et al., 2021). In forests, understory plants play an important selective role in determining the fate of tree seedlings, known as ecological filtering (George and Bazzaz, 1999a; George and Bazzaz, 1999b; Marrs and Watt, 2006; Meiners, 2014). Dense understories exacerbate the degree of light attenuation caused by the midstory and canopy (Harms et al, 2004; Royo et al., 2006), increase soil moisture (George and Bazzaz, 1999a; Nilsson and Wardle, 2005; Liu et al., 2012a) and soil carbon storage (Lyu et al., 2019). Dense understory layer also can alter animal activities such as providing shelter for some small animals or hindering animal access (Royo and Carson, 2008; Nuttle et al., 2014; Ssali et al., 2019). Ferns are one of vital components in the understory of forests and can form the recalcitrant understory layer due to their highly developed root systems, spore reproduction and cloning strategies (Page, 2002; Young and Peffer, 2010). Compared with other herbaceous understory layer, recalcitrant understory fern layer have anti-interference characteristics (e.g. drought, fire and herbivore tolerant) since developed rhizomes (Marrs and Watt, 2006; Mehltreter et al., 2010), and allelopathy characteristic (Bonanomi et al, 2006; Ismail and Chong, 2009; Kato-Noguchi et al., 2013). Thus, it can persist for long periods of time and affect tree regeneration. Previously, many studies have found that the recalcitrant fern layer can inhibit or alter seedling regeneration by changing the biotic and abiotic environment (e.g. George and Bazzaz, 1999a; George and Bazzaz, 1999b; Gallegos et al., 2015; Dietrich et al., 2017; Brock et al., 2018; Ssali et al., 2019). However, how does the effect of recalcitrant understory ferns on seedlings change with growth is still far from clear.

The responses of tree seedlings to the recalcitrant understory fern layer may vary with regeneration stage. After seedlings emerge, seedling regeneration is usually divided into the establishment and development stage (Grubb, 1977; De Steven, 1991; Kitajima et al., 2000), with seedlings at different stages having different microhabitat and resource requirements (Liu et al., 2021). The seedling establishment stage is a survival bottleneck since it fragility to many abiotic (e.g. soil texture, temperature or moisture) and biotic (predation or pathogen infection) environments (Kitajima et al., 2000; Royo and Carson, 2008; Liu et al., 2012b; Nuttle et al, 2014; Bagchi et al., 2014). After seedlings established, their survival rate will reach a relative stationary phase and transfer to growth (Grubb, 1977). As the seedlings develop, they need more resources (e.g. nutrients and light) to maintain growth (Kobe and Vriesendorp, 2011; Lin et al., 2014; Liu et al., 2017; Boonman et al., 2020). Correspondingly, the cover provided by recalcitrant understory ferns alters the microhabitat and increases resource competition between seedlings (George and Bazzaz, 1999b; Montgomery et al., 2010; Gaudio et al., 2011). For example, a dense fern understory and its litter cover changes the physical environment, influencing temperature, humidity and surface illumination (George and Bazzaz, 1999a; Liu et al., 2012a; Ssali et al., 2019). Bracken fern (*Pteridium aquilinum*) alters the soil environment creating an inorganic N-rich environment (DeLuca et al., 2013). Therefore, fern cover that generates a particular microhabitat and resource environment would change the original responses of seedling regeneration, reflected by seedling survival rate, growth rate and biomass allocation (e.g., root-shoot ratio).

Light condition is considered to be the most important abiotic factor affecting seedling regeneration (George and Bazzaz, 1999a; George and Bazzaz, 1999b; Gaudio et al., 2011; Liu et al., 2017; De Lombaerde et al., 2019; Liu et al., 2021). Many studies have found that the dense understory fern layer would greatly inhibit the regeneration of heliophile pioneer specie as the dense ferns create a low-light environment, but instead favors the regeneration of shade-tolerant tree species (Gallegos et al., 2015; Ssali et al., 2017; Brock et al., 2018; Ssali et al., 2019). For example, in a South-West Ugandan forest, Ssali et al. (2019) found that bracken (*P. aquilinum*) hinders the establishment of pioneer species but favours the germination of late-successional (more shade tolerant). In the montane forest, Bolivia, Gallegos et al. (2015) found that bracken (*P. arachnoideum*) can facilitate the seedling recruitment of Clusia and potentially other shade-tolerant tree species. Both of these studies were carried out in more open forests including a coniferous forest or disturbed forest (Gallegos et al., 2015; Dietrich et al., 2017; Ssali et al., 2019), thus the response of tree seedlings to the recalcitrant understory fern layer in closed forest is still unclear.

*D. glaucum* is one of the most widely distributed fern species throughout temperate and tropical Asia and often forms large pure colonies (Kato-Noguchi et al., 2013). It can grow up to 2 meters in height and extends the recalcitrant understory in natural forests (Song and Wang, 1995). In this study, we set fern retention and removal treatments of *D. glaucum* understory in a closed subtropical evergreen broad-leaved forest (EBLF) in eastern China. In total we transplanted 576 seedlings of 4 local dominant tree species, *Castanopsis fargesii*, *Lithocarpus glaber*, *Schima superba* and *Hovenia acerba* to the experimental treatments. We collected seedling survival, growth, and biomass data for each of the four tree species to answer two questions: (1) Whether tree seedling demography responded to the recalcitrant fern layer varied across the seedling stages? (2) Was there any species or trait-dependent effects?

## Materials and methods

### Site description

This study was conducted in the Tiantong National Forest Park in the Zhejiang Provence in eastern China (29°48′ N, 121°47′ E). This region has a subtropical monsoon climate and receives an average of 1374 mm of rainfall each year. The hot-humid-summer occurs from June to August and the cold-dry-winter occurs from December to February. Mean annual temperature is 16.2°C, with a monthly maximum temperature of 28.1 °C and minimum of 4.2 °C (Song and Wang, 1995). The soil type is Acrisol, with a medium-heavy loam texture, and the organic layer is approximately 5 cm thick, with a pH ranging from 4.4 to 5.1 (Liu et al., 2021). This region supports EBLF, in which forests are dominated by species in the Fagaceae, Theaceae and Lauraceae families (Yang et al., 2016). Due to its proximity to the Tiantong Temple, a historic site dating back more than 1,700 years, the forest has been well preserved. However, the distribution of a local fern species, *D. glaucum* gradually began to dominate the understory from the patch distribution (**Figure S1**), and persisted for 30 years, even as the forest canopy almost closed (Song and Wang, 1995; Chen et al., 2010).

### Experimental design

**Figure 1:**
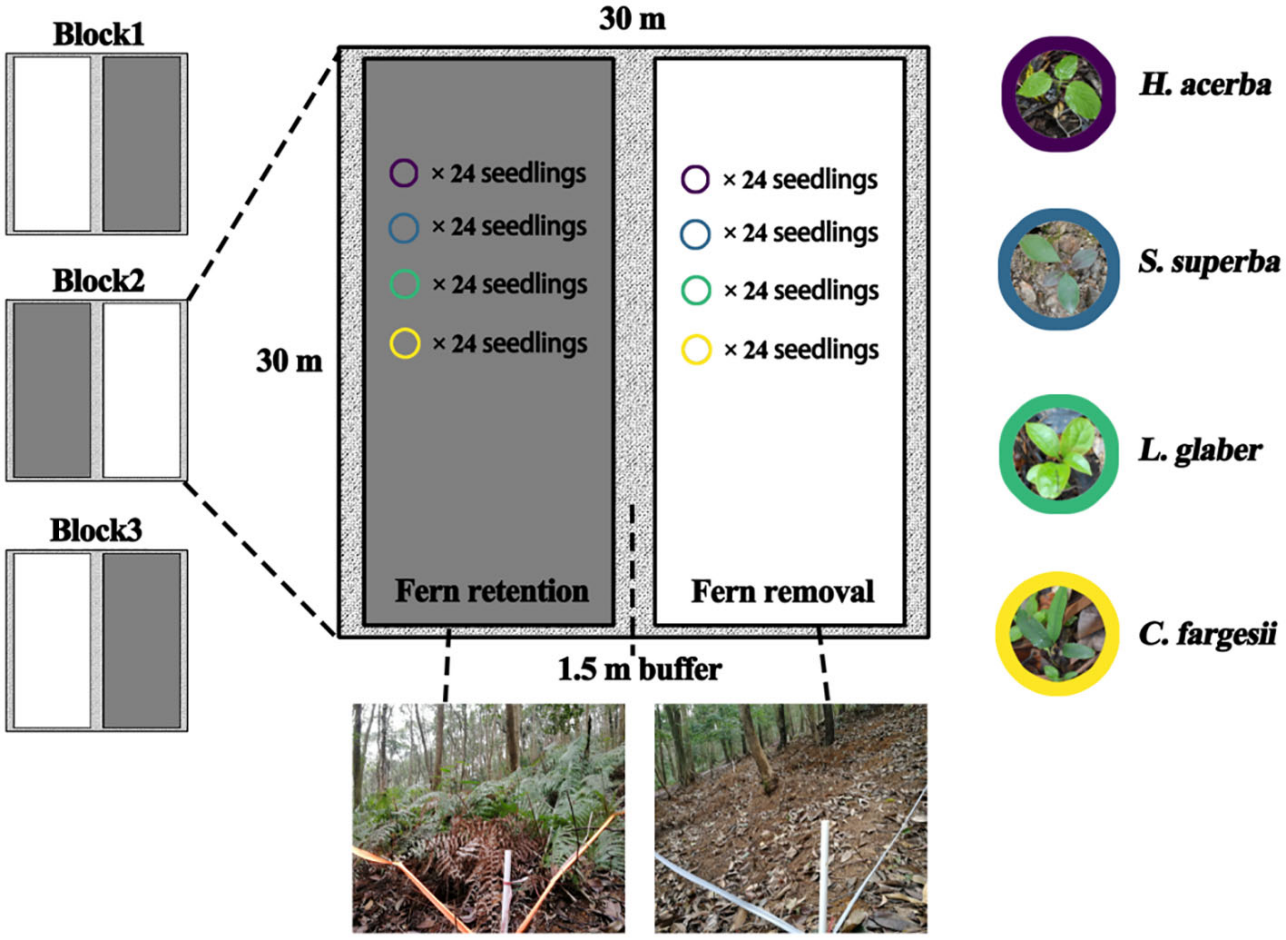
Layout of the treatments of the understory fern layer.

The field experimental site was set in a natural EBLF with a recalcitrant *D. glaucum* understory, with *D. glaucum* covering > 85% and ranging in height from 0.9 m to 1.1 m. In this experiment, we set three blocks in the similar slope position, and each block (30 m × 30 m) was split into two treatments: “Fern removal” and “Fern retention” with 1.5 m buffer area in between each treatment (**Figure 1**). Two treatments were set at parallel slope to aviod resource diffference by up and down slope. In the removal treatment, the *D. glaucum* and its litters were completely removed and other plants were kept intact. New colonized or germinated ferns were cut at regular intervals. For underground fern rhizomes, we did not remove to avoid soil disturbance. The retention treatment was not modified. All free-standing trees with diameters at breast height (DBH) ≥ 5 cm were tagged, measured and identified to the species level for all plots. The basic community and environmental information for each of the six split plots is listed in **Table S1**.

Four local dominant species were selected to test the effect of fern understory on seedling survival and growth dynamics (**Table S2**). *S. superba* (Theaceae), *L. glaber* (Fagaceae) and *C. fargesii* (Fagaceae) are the dominant tree species of EBLFs in this region and within our plots (Wang et al., 2007; Yang et al., 2011). *H. acerba* (Rhamnaceae) is a deciduous pioneer tree species, and often regenerates in forest gaps (Song and Wang, 1995). All seedlings were grown in a greenhouse. For each species, 144 healthy, uniformly sized new germinated seedlings were transplanted in June 2015 (24 seedlings × 2 split plots × 3 blocks × 4 species = 576 samples). The transplanted newly germinated seedlings of each species were distributed randomly. The transplanted seedlings were separated by 0.5 m in order to avoid the influence of each other.

### Data collection

This experiment was divided into two stages: the seedling establishment stage and seedling development stage. The field seedling establishment stage was defined as the first year after transplantation based on previous research on seedling dynamics in Tiantong National Forest Park (Liu et al., 2021). Thus, we measured the initial height of each seedling after transplantation in June 2015, and we recorded seedling survival status and measured seedling height in June 2016. In October 2017, considering as the development stage, we re-censused seedling survival status and height. Then, we took out the surviving seedlings from each plot and divided them into aboveground (stem and leaves) and underground (root) pieces. The entirety of the seedlings were dried to constant weight in 70°C and weighed.

In addition, we collected physical environmental conditions in all split plots in same time (**Table S3**).We collected leaf area index of the understory (understory LAI) and ground surface (surface LAI) in each plot by LAI-2200 (LI-COR, USA) in the center of the plot above 2 m and 0.2 m high. We collected soil temperature and water content by Em50 (METER, USA) in the summer (June) and winter (December) of 2016.

### Data analyses

To measure the seedling survival, growth, biomass accumulation, and aboveground and underground development status, we calculated the survival rate (*P_survival_*) and relative growth rate (*RGR*) of each species’ seedlings in both the establishment and development stages, and total biomass (*B_total_*) and root shoot ratio (*R_r/s_*) of alive seedlings at the end of experiment (Poorter et al., 2012) using the following formulas:


(1) $$P_{survival}=N_{i}/N_{i-1}$$

Where *P_survival_* is the seedlings survival rate in the plot, *Ni* is the number of living seedlings in *i*th census in the plot; *N_i-1_* in (*i-1*)th census in the plot.


(2) $$RGR=(\ln H_{i}-\ln H_{i-1})/(T_{i}-T_{i-1})$$

Where *RGR* is the relative growth rate of living seedlings in the plot (Liu et al., 2017). *H_i_* is height of seedling in *i* th census in the plot; *H_i-1_* in (*i-1*) th census in the plot. *T_i_* is number of months from *i* th census to seedling transplanted; *T_i-1_* from (*i-1*)th census.


(3) $$B_{total}=B_{aboveground}+B_{underground}$$

(4) $$R_{r/s}=B_{underground}{/B}_{aboveground}$$

Where *B_total_* and *R_r/s_* are the total biomass and root shoot ratio of living seedlings at the end of the experiment. *B_abovegorund_* is the biomass of aboveground living seedlings; *B_underground_* belowground.

To estimate the effect of the recalcitrant *D. glaucum* understory on seedling survival, we built generalized liner mixed-effects models (GLMMs) with binomial errors for transplanted seedlings of each species in the establishment and development stages. Due to location of experiment in three random blocks, we set blocks and its containing plots as random parts of the GLMMs. Additionally, light condition and initial height of seedlings influences seedling survival (Comita and Hubbell, 2009; Lin et al., 2014), thus in addition to the explanatory variable of treatment method, each GLMM included understory LAI of each split plot and initial height of each seedling as explanatory variables. The utilized model with random effects can be specified as:


(5) $${\text{Y}}_{\text{ijk}}\sim binomial(1,\pi_{\text{ijk}})$$

(6) $$logit(\pi_{\text{ijk}})={\left[\alpha +\beta_{1}\times x_{Fern}+\beta_{2}\times x_{H\text{eight}.L}+\beta_{3}\times x_{LAI.U}\right]}_{\text{fixed.part}}+{\left[\mu_{\alpha |j\text{/k}}+\mu_{\alpha \text{|k}}\right]}_{\text{random.part}}$$

Where *Y_ijk_* is 1 if seedling *i* is alive in the plot *j* of block *k* and 0 otherwise, *π_ijk_* is the survival probability of focal seedling (equation 5). In the fixed part, *α* and *β* refer to an intercept and a vector of coefficients of explanatory variables *x*, respectively. *x_Fern_* indicates the explanatory variables of fern retention vs fern removal treatments for the recalcitrant *D. glaucum* understory. *x_Height.L_* and *x_LAI.U_* indicate the explanatory variables of log-transformed height of seedling *i* in last census and LAI of plot *j* in the understory. The random part has two levels, first level is *α* with random effect within each split plot *j* belonging to block *k* and seconds within block *k* (equation 6).

To estimate the effect of recalcitrant *D. glaucum* understory on living seedling growth, we built liner mixed-effects models (LMMs) for living seedlings of each species in the establishment and development stages. The fixed and random portions of these LMMs are the same as in equation 6. The model with the random effects can be specified as:


(7) $${\text{RGR}}_{\text{ijk}}={\left[\alpha +\beta_{1}\times x_{Fern}+\beta_{2}\times x_{H\text{eight}.L}+\beta_{3}\times x_{LAI.U}\right]}_{\text{fixed.part}}+{\left[\mu_{\alpha |j\text{/k}}+\mu_{\alpha \text{|k}}\right]}_{\text{random.part}}$$

Where *RGR_ijk_* is relative growth rate of seedling *i* in plot *j* of block *k*.

To estimate the effect of recalcitrant *D. glaucum* understory on biomass accumulation and aboveground/underground growth pattern of living seedlings, we also built LMMs similar to equation 7, with the dependent variables as biomass (*B_ijk_*) and root shoot ratio (*R_ijk_*) of alive seedling.

All the continuous variables were normalized by subtracting the mean of the variable and dividing by the standard deviation. All analyses were conducted in R 4.1.1 (R Development Core Team, 2021). The GLMMs and LMMs were fit using the “glmer” and “lmer” functions of “lme4 1.1–13” package (Bates et al., 2013).

## Results

### Seedling survival between fern retention and removal

**Figure 2:**
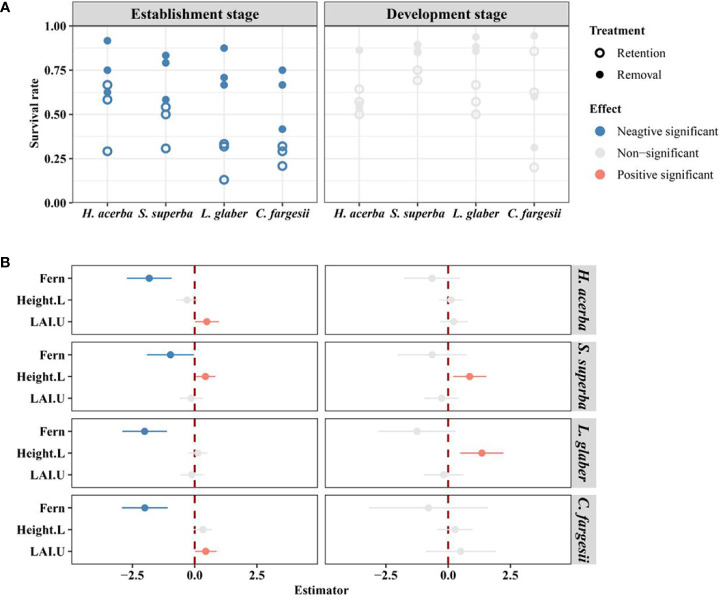
The difference in survival rate of seedlings between retention and removal treatments in establishment and development stages **(A)**, and the corresponding estimator of explanatory variables in generalized linear mixed-effects models **(B)**. Fern in B refers to the effect of retention treatment vs removal treatment; Height.L refers to the log-transformed height of seedling in the last census; LAI.U is the leaf area index of the plot in the understory. The error bars in B represent 1.96*se around estimator in generalized linear mixed models. Blue and red points in B indicate parameter estimates significantly different from zero at the alpha = 0.05 level.

After 28-months of monitoring, *L. glaber* had the highest seedling survival rate (66.7%) in the removal treatment, followed by *S. superba* (63.9%), *H. acerba* (51.4%) and *C. fargesii* (38.9%). In the retention treatment, *S. superba* had the highest survival rate (32.7%), then *H. acerba* (29.2%), *C. fargesii* (16.4%), and finally *L. glaber* (14.5%) (**Table S4**). In the establishment stage, the seedling survival rate of all species in the removal treatment was significantly higher than that of those in the retention treatment (**Figure 2A**). In the development stage, there was no significant difference in seedling survival rate between the two treatments for all four species (**Figure 2A**). Additionally, the seedling initial height of *S. superba* had a significant positive effect (*P*<0.05) on seedling survival in both the establishment and development stages, while the effect of seedling initial height of *L. glaber* was only significant in the development stage (*P*<0.01) (**Figure 2B**). Meanwhile, the seedling survival rate of *C. fargesii* and *H. acerba* in establishment stage increased with increasing understory LAI (LAI.U) (**Figure 2B**).

### Seedling growth between fern retention and removal

**Figure 3:**
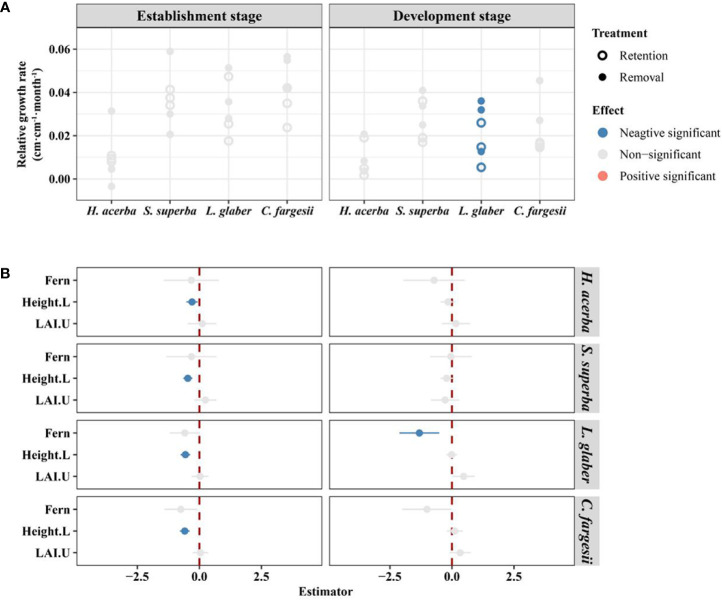
The difference in relative growth rate of living seedlings between retention and removal treatments in establishment and development stages **(A)**, and the corresponding estimator of explanatory variables in linear mixed-effects models **(B)**. Fern in B refers to the effect of retention treatment vs removal treatment; Height.L refers to the log-transformed height of seedling in the last census; LAI.U is the leaf area index of the plot in the understory. The error bars in B represent 1.96*se around estimator in linear mixed models. Blue and red points in B indicate parameter estimates significantly different from zero at the alpha = 0.05 level.

In the establishment stage, there were no significant difference in seedling relative growth rate between the two treatments for all four species (**Figure 3A**), while initial height of seedlings had a significant negative effect on seedling relative growth rate in all four species (**Figure 3B**). In the development stage, *L. glaber*’s seedling relative growth rate in the retention treatment was significantly lower than in the removal treatment (*P*<0.05) (**Figure 3A**).

### Seedling biomass allocation between fern retention and removal

**Figure 4:**
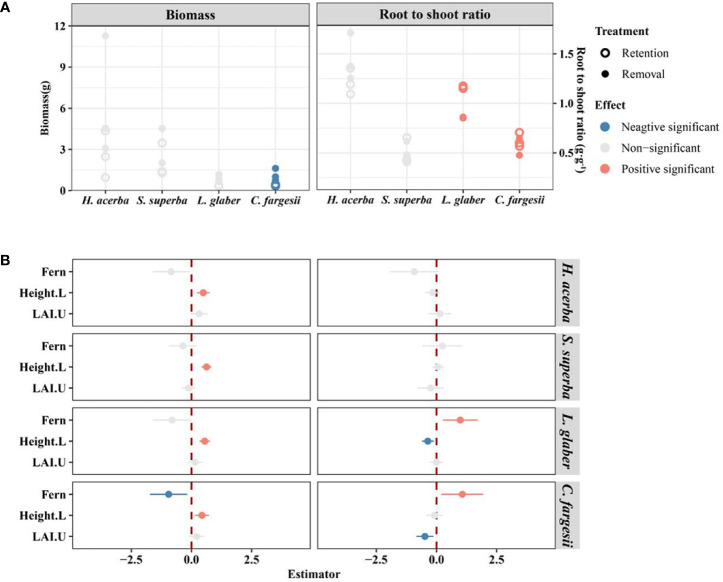
The difference in biomass and root/shoot ratio of living seedlings between retention and removal treatments in establishment and development stages **(A)**, and the corresponding estimator of explanatory variables in linear mixed-effects models **(B)**. Fern in B refers to the effect of retention treatment vs removal treatment; Height.L refers to the log-transformed height of seedling in the last census; LAI.U is the leaf area index of the plot in the understory. The error bars in B represent 1.96*se around estimator in linear mixed models. Blue and red points in B indicate parameter estimates significantly different from zero at the alpha = 0.05 level.

The seedling biomass of *C. fargesii* in the retention treatment was significantly lower than in the removal treatment (*P*<0.05), while the initial height of seedlings had a significant positive effect on seedling biomass in all four species (**Figure 4A**). The root shoot ratio of *C. fargesii* and *L. glaber* was significantly higher in the retention treatment than in the removal treatment (*P*<0.05) (**Figure 4**), while, the root shoot ratio of *C. fargesii* decreased with understory LAI (**Figure 4B**).

## Discussion

The recalcitrant fern layer strongly inhibited tree seedling regeneration in our subtropical forest. Our study showed that fern retention significantly inhibited the seedling survival of all four species in establishment stage but not for development stage. All four species seedlings grew better in plots where ferns were removed than in plots where ferns were present, but the intensity varied among species. Specially, the significant results of RGR, total biomass or root-to-shoot ratio were found in both shade tolerant species *C. fargesii* and *L. glaber.*

The response of seedling regeneration to the recalcitrant understory fern layer differed among regeneration stages. At the establishment stage, the seedling survival of all four species was significantly inhibited by the *D. glaucum* layer, and there was no significant difference in the RGR of each species between the retention and removal treatments (**Figures 2**, **3**). This result demonstrates the consistent effect of *D. glaucum* understory on different tree species seedlings. In general, because seedlings in the establishment stage are extremely sensitive to microhabitat (Gilbert et al., 2001; Murphy et al., 2017; Kuang et al., 2017; Liu et al., 2021), newly germinated seedlings suffer the highest mortality during establishment (Murphy et al., 2017; Kuang et al., 2017), and only a fraction of seedlings can establish through this demographic bottleneck for populations (De Steven, 1991; Gurevitch et al., 2006). Furthermore, the *D. glaucum* layer formed a disturbed microhabitat which further intensified the demographic bottleneck (**Figure S1**) due to the sensitivity of newly germinated seedlings to microhabitat changes (Royo et al., 2006; Gallegos et al., 2015). In our study, the *D. glaucum* layer significant changed the soil temperature in the winter, the water content in the summer, and especially surface light in both seasons (See **Table S3**). The fern understory reduced light levels with surface LAI equaling 3.84 in the removal treatment compared to 9.13 in the retention treatment (**Table S3**). Other research has shown that light availability has a significant positive effect on early-stage seedling survival (Liu et al., 2021). Therefore, we conclude that the large environmental variations caused by the fern layer limited seedling survivals for all tree species.

In contrast, at the development stage, the fern layer did not significantly inhibit the survival rates of all of transplant species, but it did significantly effect the RGR of *L. glaber* (**Figures 2**, **3**). As seedlings establish, seedlings may have adapted to the existing environment and seedling mortality stabilized (**Figure S2**). Seedlings move to the development stage by absorbing above- and below ground resources (Grubb, 1977). Due to the different effects of resource competition with the fern layer, the filtering effect of the fern layer will be reflected in the difference in the seedling relative growth rate in the development stage (George and Bazzaz, 1999b; Strengbom et al., 2004; Song et al., 2012; De Lombaerde et al., 2019). According to our results, seedlings showed different reflections under the recalcitrant fern layer between the establishment and development stage.

It is worth to mentioning that initial height had a significant influence on some species’ seedling growth and survival (**Figures 2**, **3**). It is generally believed that taller established seedlings can obtain more light resources and thus have a growth advantage (Liu et al., 2017). However, in our study, only two species showed a significant positive effect of height on seedling survival at the development stage, and, opposingly 4 species show a negative effect of height on seedling RGR at the establishment stage. This may indicate that survival is most important for seedlings during the establishment stage because more resources are allocated to survival with a high initial height that actually reduces the relative growth rate. These results demonstrate the indirect temporal differentiation of the filtering effect on seedling regeneration.

Environmental stress of the recalcitrant understory fern alters the seedling biomass allocation of tree species. In our study, alive seedlings of two Fagaceae evergreen species, *L. glabe* and *C. fargesii*, were more influenced by ferns with the root shoot ratio significantly higher in the retention treatment than in removal treatment (**Figure 4**), meaning more biomass was allocated to the roots. According to the “balanced growth hypothesis” (Shipley and Meziane, 2002), plants will allocate relatively more biomass to roots if the limiting factor for growth is below ground (e.g. nutrients, water), whereas they will allocate relatively more biomass to shoots if the limiting factor is above ground (e.g. light) (Poorter et al., 2012). Therefore, our results suggests that *D. glaucum* may affect alive seedling growth more through subsurface competition than above ground light interception in our study area.

Our research results are consistent findings that show the recalcitrant understory fern layer acts as an ecological filter (George and Bazzaz, 1999a; George and Bazzaz, 1999b; Royo et al., 2006; Wright et al., 2012; Ssali et al., 2019; Beltran et al., 2020). For example, the reduced survival rate of two treatments of *L. glaber* was 2.3 times that of *H. acerba* (**Table S4** and **Figure 2**) and the RGR of *L. glaber* seedlings in the development stage had a significant negative effect under the fern treatment but this was not the case for other species(**Figure 3**). However, from our results on relative growth rate (*L. glabe*, **Figure 3**), total biomass (*C. fargesii*, **Figure 4**), root shoot ratio (*L. glabe* and *C. fargesiii*, **Figure 4**) and seedling height (*L. glabe* and *C. fargesii*, **Table S5**), two shade tolerant species, ferns showed stronger inhibiting effects on *L. glabe* and *C. fargesii* than on the two shade intolerant or moderate species which is inconsistent with previous studies of bracken (Gallegos et al., 2015; Ssali et al., 2019). These studies suggest that bracken ameliorates contain harsh abiotic conditions which increasing the probability of shade-tolerant tree species’ seedling recruitment because brackens are dominat in areas degraded by fires (Gallegos et al., 2015) or by mixed disturbance (Ssali et al., 2019). In contrast, the recalcitrant *D. glaucum* layer in our study is distributed in closed forests (more than 778 individuals per hectare with mean dbh from 14.9 cm -19.1 cm, see **Table S2**). Accordingly, the dense fern layer may not ameliorate, but instead worsen the suitability of shade-tolerant species *L. glabe* and *C. fargesii*. For shade intolerant or moderate species, the environment under the closed canopy is not suitable no matter whether ferns are distributed or not, leading to no significant difference between the retention and removal treatments. Therefore, because shade-tolerant species was inhibited by the fern layer and shade-intolerant species are not inherently adapted to the environment of closed forests, natural regeneration would be extremely difficult and requires artificial forest management.

## Conclusion

Understanding the effect of the recalcitrant understory fern on natural regeneration requires thorough knowledge of how tree seedlings will respond at different times and growth parameters. Our study provides evidence of the ecological filtering effect of a recalcitrant understory fern, *D. glaucum*, in a subtropical forest. Furthermore, the ecological filter effect on a species can vary between seedling regeneration stages, but the seedling survival for all species is inhibited significantly during establishment stage with some species showing significant lower relative growth rates in the fern retention area. Future studies should include more regeneration stages such as seed dispersal, seed germination for fully understanding the influence of recalcitrant understory fern layer on the forest renewal process. Moreover, shade tolerant tree species were more inhibited by fern disturbed areas in closed forest. From the perspective of forest health and management, we need to take measures to curb forest fern outbreaks which would help unblock the forest regeneration process.

## Data availability statement

The raw data supporting the conclusions of this article will be made available by the authors, without undue reservation.

## Author contributions

QY, XW and HL conceived and designed the study. QY, HL, ML and ZZ collected the data. ML and HL provided analysis tools and analyzed the data. QY, HL, JZ and GS drafted and revised the article. All authors agree to be accountable for all aspects of the work. All authors contributed to the article and approved the submitted version.

## Funding

This study was financially supported by the National Natural Science Foundation of China (Grant No. 31901103 and 31800351) and by “Fundamental Research Funds for the Central Universities”.

## Acknowledgments

We thank Qingkai Lin, Siyuan Ren, Chunhui Ma and Mingjie Xu for help with the field work. We are also grateful to professor Enron Yan for his constructive comments on this study. We would like to thank native speaker from the HighEdit Company for assistance with English language editing of this manuscript.

## Conflict of interest

The authors declare that the research was conducted in the absence of any commercial or financial relationships that could be construed as a potential conflict of interest.

## Publisher’s note

All claims expressed in this article are solely those of the authors and do not necessarily represent those of their affiliated organizations, or those of the publisher, the editors and the reviewers. Any product that may be evaluated in this article, or claim that may be made by its manufacturer, is not guaranteed or endorsed by the publisher.
