# Supplementary material for: Responses of tree seedlings to understory filtering by the recalcitrant fern layer in a subtropical forest
Source: Front Plant Sci. 2022 Nov 24;13:1033731. doi: 10.3389/fpls.2022.1033731 (PMC9730283; doi:10.3389/fpls.2022.1033731)
Supplement: Supplementary file 1 [file DataSheet_1.docx]

**Supporting information**

| Block | Treatment | Area/m^2^ | Altitude /m | Slope /(°) | Aspect | dominant species | Richness of trees | Mean DBH of tree± SE /cm | Mean height of tree± SE /m | Density of tree /ha |
| --- | --- | --- | --- | --- | --- | --- | --- | --- | --- | --- |
| 1 | Retention | 450 | 102 | 13 | Southeast | *Schima superba* | 8 | 18.8±1.4 | 12.6±0.5 | 956 |
|  | Removal | 450 | 102 | 13 | Southeast | *Schima superba* | 8 | 19.0±1.2 | 12.4±0.5 | 889 |
| 2 | Retention | 450 | 86 | 15 | Southeast | *Schima superba* | 12 | 19.1±1.8 | 11.3±1.0 | 844 |
|  | Removal | 450 | 86 | 15 | Southeast | *Schima superba* | 11 | 17.4±2.3 | 10.6±1.1 | 778 |
| 3 | Retention | 450 | 114 | 13 | Southeast | *Schima superba* | 9 | 17.3±0.9 | 12.1±0.5 | 1022 |
|  | Removal | 450 | 114 | 13 | Southeast | *Schima superba* | 10 | 14.9±0.8 | 11.1±0.6 | 1178 |

Table S1 The basic community and environmental information of 6 split plots

Table S2 Basic information of four selected tree species

| Species | Genus | Family | Life form | Succession stage |
| --- | --- | --- | --- | --- |
| *Hovenia acerba* | *Hovenia* | Rhamnaceae | Deciduous species, shade-intolerance | Early succession |
| *Schima superba* | *Schima* | Theaceae | Evergreen species, moderate | Middle and late succession |
| *Lithocarpus glabe* | *Lithocarpus* | Fagaceae | Evergreen species, shade-tolerance | Middle and late succession |
| *Castanopsis fargesii* | *Castanopsis* | Fagaceae | Evergreen species, shade-tolerance | Late succession |

Table S3 Physical environments of fern retention and removal treatments

| Treatment | Surface LAI | | Understory LAI | | Soil temperature in summer (℃) | | Soil temperature in winter(℃) | | Soil water content in summer (%) | | Soil water content in winter (%) | |
| --- | --- | --- | --- | --- | --- | --- | --- | --- | --- | --- | --- | --- |
|  | Mean value ± SE | *P* value | Mean value ± SE | *P* value | Mean value ± SE | *P* value | Mean value ± SE | *P* value | Mean value ± SE | *P* value | Mean value ± SE | *P* value |
| Retention | 9.13±0.19 | <0.001 | 3.92±0.28 | 0.256 | 23.57±0.15 | 0.067 | 14.20±0.10 | 0.009 | 30.67±1.45 | 0.049 | 32.33±1.33 | 0.630 |
| Removal | 3.84±0.14 |  | 3.47±0.18 |  | 24.27±0.24 |  | 13.10±0.21 |  | 37.67±2.03 |  | 33.67±2.19 |  |

*Note*: *P* value refers to the difference between fern retention and removal treatments through variance analysis

Table S4 Final survival rate and relative growth rate at the end of experiment between fern retention and removal treatments

| Species | Survival rate ± SE | |  | Relative growth rate ± SE(cm·cm^-1^·month^-1^) | |
| --- | --- | --- | --- | --- | --- |
|  | Retention | Removal |  | Retention | Removal |
| *C. fargesii* | 0.164±0.063 | 0.389±0.160 |  | 0.023±0.004 | 0.044±0.005 |
| *L. glaber* | 0.145±0.030 | 0.667±0.042 |  | 0.022±0.006 | 0.033±0.002 |
| *S. superba* | 0.327±0.048 | 0.639±0.069 |  | 0.034±0.005 | 0.036±0.005 |
| *H. acerba* | 0.292±0.064 | 0.514±0.141 |  | 0.009±0.002 | 0.019±0.006 |

Table S5 Final height at the end of experiment between retention and removal treatments

| Species | Treatment | Mean of height ± SE | Estimator | *P*-value |
| --- | --- | --- | --- | --- |
| *H.acerba* | Retention | 41.85±17.56 | -0.725 | 0.204 |
|  | Removal | 45.31±9.51 |  |  |
| *S.superba* | Retention | 21.63±8.35 | -0.159 | 0.753 |
|  | Removal | 23.02±5.81 |  |  |
| *L.glaber* | Retention | 8.10±2.28 | -0.735 | 0.0229 |
|  | Removal | 11.03±2.07 |  |  |
| *C.fargesii* | Retention | 7.59±1.39 | -0.952 | 0.099 |
|  | Removal | 14.13±5.88 |  |  |

Notes: Estimator and *P*-value refers to the effect of fern retention treatment vs fern removal treatment on final height through liner mixed-effects models with random effects within each plot in corresponding block.

Table S6 The standard deviation of random effects in generalized linear mixed-effects models and linear mixed-effects models

| **Dependent variable** | **Regeneration stage** | **Species** | **σ_block_** | **σ_plot/block_** |
| --- | --- | --- | --- | --- |
| Survival rate | Establishment stage | *H. acerba* | 0 | 0 |
|  |  | *S. superba* | 1.149×10^-5^ | 0.2044 |
|  |  | *L. glaber* | 0 | 2.705×10^-8^ |
|  |  | *C. fargesii* | 0 | 0 |
|  | Development stage | *H. acerba* | 0 | 0.1451 |
|  |  | *S. superba* | 0 | 0 |
|  |  | *L. glaber* | 0 | 0 |
|  |  | *C. fargesii* | 0.7425 | 0.5852 |
| Relative growth rate | Establishment stage | *H. acerba* | 2.334×10^-5^ | 0.5032 |
|  |  | *S. superba* | 0 | 0.4810 |
|  |  | *L. glaber* | 0.2656 | 0 |
|  |  | *C. fargesii* | 1.512×10^-5^ | 0.2261 |
|  | Development stage | *H. acerba* | 0 | 0.48 |
|  |  | *S. superba* | 0.6425 | 0 |
|  |  | *L. glaber* | 0.3787 | 0 |
|  |  | *C. fargesii* | 0 | 0.3 |
| Biomass | / | *H. acerba* | 0 | 0.2056 |
|  |  | *S. superba* | 0 | 0.1897 |
|  |  | *L. glaber* | 0 | 0.2080 |
|  |  | *C. fargesii* | 0 | 0 |
| Root/shoot ratio | / | *H. acerba* | 0 | 0.3263 |
|  |  | *S. superba* | 0.5642 | 0 |
|  |  | *L. glaber* | 0.07383 | 0 |
|  |  | *C. fargesii* | 0 | 0 |


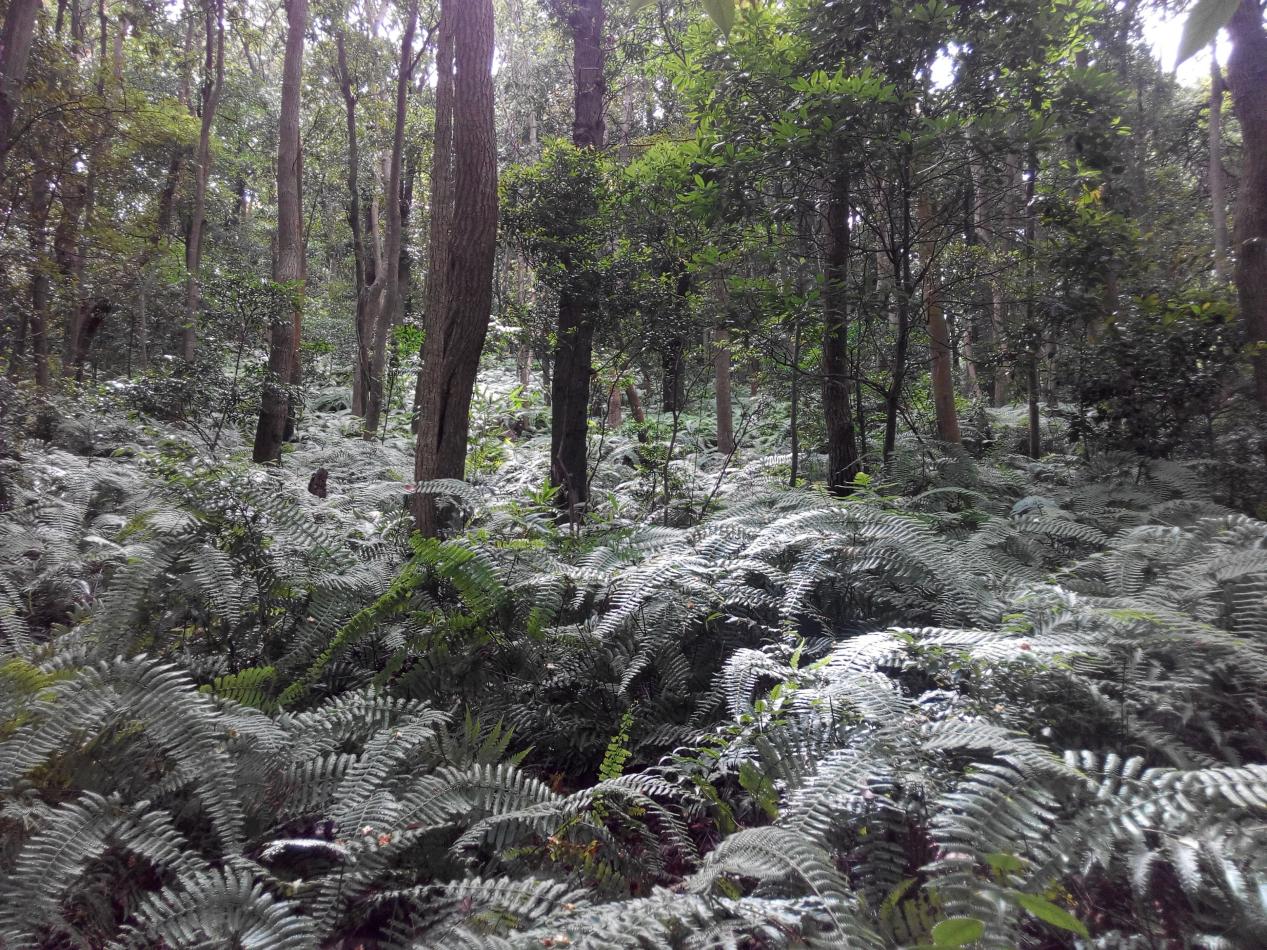


Figure S1 Photographic example with *Diplopterygium glaucum* fern in subtropical evergreen broad-leaved forest in our study.


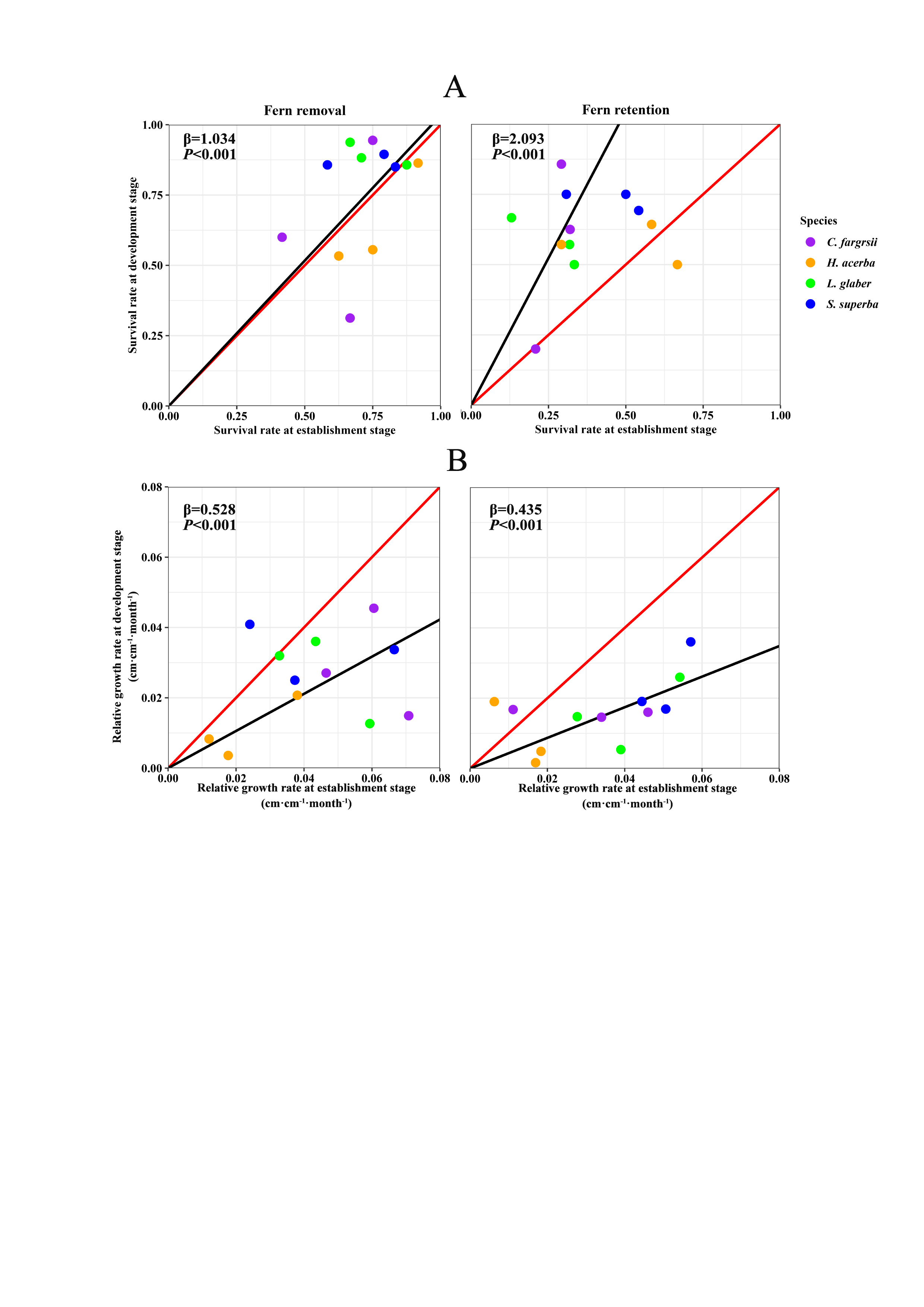
Figure S2 Correlation of survival (A) and growth (B) status of seedlings between the establishment and development stage in fern retention and removal treatments. $\beta$ refer to a vector of coefficients in liner mixed-effects models with random effect within species. The black lines represent the fitted regression line by linear mixed-effects models. The red lines represent standard line of α=0 and β = 1.
